# Complications of the Percutaneous Mitral Valve Edge-To-Edge Repair: Role of Transesophageal Echocardiography

**DOI:** 10.3390/jcm11164747

**Published:** 2022-08-14

**Authors:** Guisela Flores, Dolores Mesa, Soledad Ojeda, Javier Suárez de Lezo, Rafael Gonzalez-Manzanares, Guillermo Dueñas, Manuel Pan

**Affiliations:** 1 Cardiology Department, Reina Sofia University Hospital, 14004 Cordoba, Spain; 2 Maimonides Institute for Research in Biomedicine of Cordoba (IMIBIC), 14004 Cordoba, Spain; 3 Faculty of Medicine and Nursing, University of Cordoba, 14004 Cordoba, Spain

**Keywords:** mitral regurgitation, complications, edge-to-edge repair

## Abstract

The use of transcatheter edge-to-edge repair for the treatment of mitral regurgitation has markedly increased in the last few years. The rate of adverse events related to the procedure is low; however, some of the complications that may occur are potentially dangerous. Due to the growing popularity of the technique, which is no longer limited to high-volume centers, knowledge of the complications related to the procedure is fundamental. Transesophageal echocardiography has a key role in the guidance of the intervention while allowing for the avoidance of most of these adverse events, as well as enabling us to diagnose them early. In this article, we review the main complications that might present during a transcatheter mitral edge-to-edge repair procedure (tamponade, thromboembolic events, single leaflet device attachment, device embolization, vascular injury…) while highlighting key aspects of transesophageal echocardiographic monitoring in the prevention and prompt diagnosis of these complications.

## 1. Introduction

Mitral regurgitation (MR) is a prevalent valve disease with high mortality and morbidity. Percutaneous mitral valve repair using transcatheter edge-to-edge repair (TEER) has been shown to be safe and effective for the treatment of mitral regurgitation. Multiple randomized controlled trials and retrospective registries have shown low complication rates, with a relevant decrease in major adverse events from 15% in 2005 to less than 3.5% in 2020 [1] (Table 1). Nevertheless, TEER is not exempt from potential complications, making a meticulous patient selection and an adequate timing of the procedure primordial to prevent increased risk.

Transesophageal echocardiography (TEE) plays a fundamental role in minimizing the risk of procedural complications, as well as in their early detection [3]. Some authors have also demonstrated the superiority of real-time 3-D transesophageal echocardiography (3D-TEE) not only in mitral clip guidance but also in the early detection of complications [4]. There is limited experience with intracardiac echocardiography, but, in the case of contraindication to TEE, it could be an alternative [5].

In this review, we cover the main complications that may occur during mitral valve TEER, with a special focus on the usefulness of intraprocedural TEE in the management of these potentially life-threatening conditions. Individual cases with practical tips and detailed images are provided throughout the text. 

## 2. Pericardial Effusion/Tamponade

Pericardial effusion or tamponade are generally caused by an erroneous transeptal puncture; nevertheless, these are rare complications, with a described incidence of 2.5–3% in the initial phase of the learning curve, considerably decreasing to 0–0.5% in relation to higher implanter experience and the consequent use of 3D-TEE guiding [6]. A higher incidence can be expected in challenging cases with a thick or very floppy septum, post-surgery septum, or patients with chest wall deformities [2].

**Figure 1:**
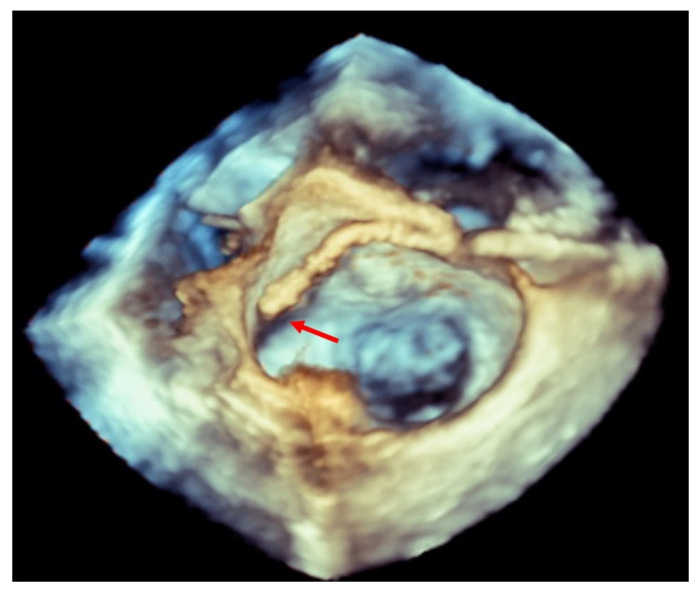
Three-dimensional transesophageal echocardiogram showing a clip inside the left atrium touching the lateral wall (red arrow).

Transesophageal echocardiographically guided transseptal puncture aiming to achieve a posterior and superior position is the main key to avoiding potential complications in this procedure [6]. The use of 3D-TEE guiding has decreased the incidence of this complication, as well as the possibility of eroding the walls of the left atrium and neighboring structures with catheters and devices [2,7] (Figure 1).

## 3. Thromboembolic Events

During mitral valve TEER, potentially thrombogenic materials are introduced through the venous system and across the transeptal access to the left atrium and ventricle. These materials, as well as the maneuvers required for clip implantation, involve potential thromboembolic events. Data registered in several reviews demonstrate that major adverse cardiac and cerebrovascular events have a low incidence (3–7%). The in-hospital myocardial infarction and postprocedural stroke rate ranges from 0 to 3% [7,8,9,10].

**Figure 2:**
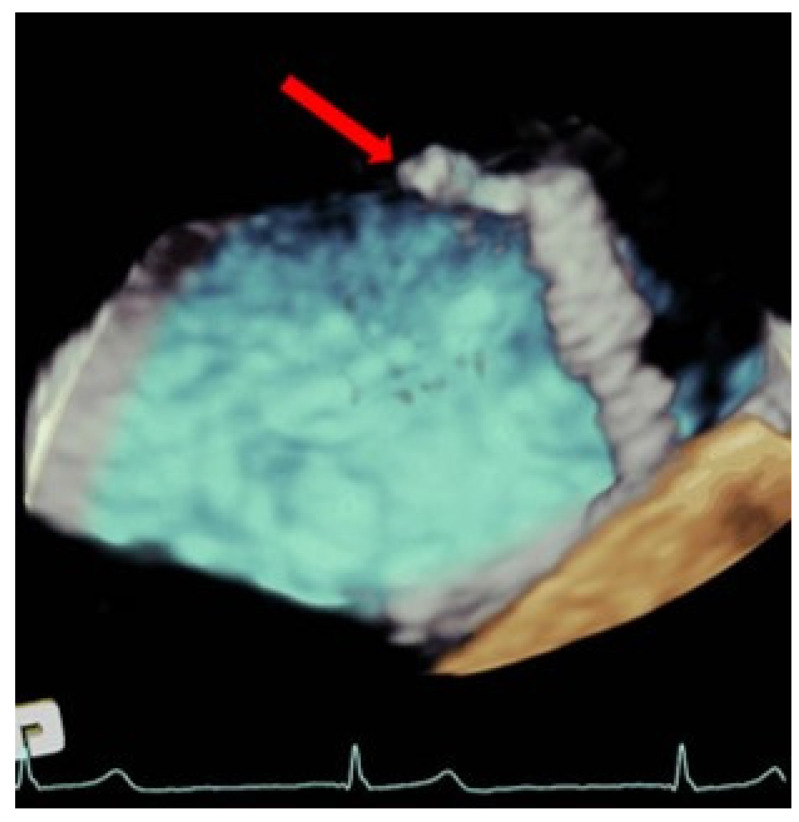
Three-dimensional transesophageal echocardiogram TEE image showing a large thrombus (red arrow) at the tip of the clip that is emerging from the delivery catheter within the left atrium.

Even though the rate of thromboembolic events is anecdotic and usually multifactorial, thrombus forming in the delivery system can have devastating consequences and should be prevented by targeting a high level of anticoagulation (with an activated clotting time between 250 and 300 s) and by constantly flushing the delivery system. Some cases of post-procedural thrombus formation in the left chambers have been reported, probably related to blood stasis after the disappearance of the mitral regurgitation jet and with long procedures [11,12]. TEE is very useful in the early detection of thrombi adhered to catheters, which allows us to act quickly by withdrawing them by intensifying anticoagulation. (Figure 2). 

## 4. Device-Related Complications

Device-related complications can be classified as functional device failure (such as persistent MR or mitral stenosis) or structural device failure, including clip detachment (partial or complete with possible clip embolization), injury of mitral leaflets, or subvalvular apparatus [1]. TEE imaging after mitral valve clip intervention includes a careful evaluation of device integrity, position, stability, and interaction with adjacent structures.

### 4.1. Functional Device Failure

The success of a TEER procedure can be defined as a reduction in the MR to be no greater than mild, trying to reach a trace or absent MR. Although, at first, the inclusion criteria for TEER were more restrictive, as the number of experienced centers has grown, more cases with challenging anatomy are performed and MR reduction can also be considered acceptable when post-procedure MR is reduced by at least one grade from the baseline and to no more than moderate (2+) in severity [13]. From the third generation onwards, the Mitraclip^®^ (Abbott Park, IL, USA) device was improved to treat patients with complex mitral anatomies, such as longer, redundant, or restricted leaflet and large flail [6]. 

#### 4.1.1. Persistent Mitral Regurgitation

Persistent MR is an important prognostic factor for both mortality and rehospitalization in the follow-up of patients with mitral clip implantation [9,14]. Undoubtedly, TEE is the technique that allows for the real-time assessment of residual mitral regurgitation after clip implantation, allowing for grasping optimization, as well as the implantation of more than one device to achieve the best possible result with minimal residual regurgitation [2]. An accurate quantification of residual MR is of great importance; however, this is difficult since, after the implantation of one or several clips, the mitral valve became a valve with two or more orifices and several residual and often eccentric jets tend to remain. A recently published guideline provides complete information about the quantification focus on the fact that the evaluation of residual MR requires the careful integration of multiple parameters, as no single parameter is sufficiently accurate to assess the MR severity post-clip. In addition, it is important to compare post-procedural parameters to preprocedural baseline images and to evaluate them under the same hemodynamic conditions, which can help to evaluate the change in MR severity [15]. Three-dimensional TEE was graded as superior to 2D-TEE for the quantification of residual mitral regurgitation and the position relative to the residual regurgitant jet after clip arm closure [13]. Despite 3D vena contracta assessment being useful according to some authors [16,17], it is currently not fully validated.

#### 4.1.2. Mitral Stenosis

In an attempt to achieve a greater reduction in the MR, a higher transmitral valve gradient can be induced. Recently, reaching a mean transvalvular gradient pressure > 5 mmHg has been associated with adverse clinical and functional outcomes in patients with degenerative MR, but not in patients with functional MR [18]. Real-life information regarding mitral stenosis in this scenario is scarce because most studies only report the mean transmitral gradient but are missing other parameters about relevant mitral stenosis [6]. TRAMI registry is the only one reporting the rate of relevant mitral stenosis, which was lower than 1% [7].

**Figure 3:**
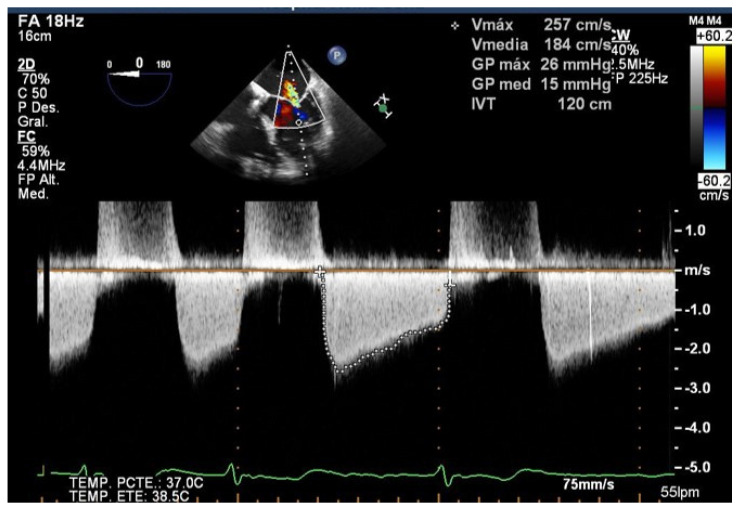
Severe mitral stenosis after implantation of a mitral clip measured by Doppler echo.

TEE allows us to study the basal mitral area (which must be at least more than 4 cm^2^), and the mobility, flexibility, and thickness of the leaflets in order to avoid significant mitral stenosis when one or more clips are implanted. Likewise, TEE is essential in the evaluation of the appearance of mitral stenosis after grasping. Medium gradients greater than 5 mmHg are not acceptable since they have been related to worse outcomes [19] (Figure 3). When this happens, the grasping must be carried out again to try to avoid the stenosis, and the implant must be abandoned when this is not possible. 

### 4.2. Structural Device Failure

#### 4.2.1. Single Leaflet Device Attachment

Single leaflet or partial clip detachment, most commonly referred to as single leaflet device attachment (SLDA), is the most frequent complication, ranging from 0 to 4.8% in the available registries. It can occur during the procedure (acute), the first days after the procedure (subacute), or it might be discovered during the follow-up (late). Most cases happen during the procedure and can be resolved by implanting a second device to stabilize the first one [2]. Regarding the mechanism, it is assumed that SLDA is due to insufficient leaflet grasping, while SLDA after adequate grasping is typically caused by leaflet tear or perforation due to poor tissue quality. The majority of SLDA involves detachment from the posterior mitral valve leaflet. Even though thefourth generation Mitraclip^®^ device allows for independent leaflet capture, reducing the risk of insufficient leaflet grasping, it does not reduce the risk of perforation or leaflet tearing. The use of wider G4 devices (XTW and NTW) may not imply a reduction in the risk of SLDA but may induce higher transmitral gradients. For this reason, a second wide clip implantation should be avoided if there are mild to moderate gradients after the first one.

**Figure 4:**
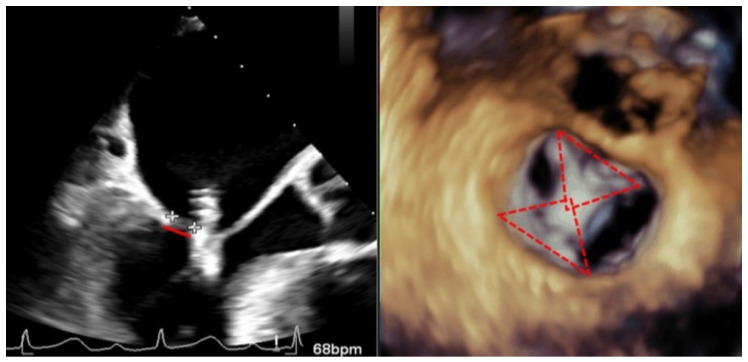
Transesophageal echocardiogram during grasping. (**Left**) Two-dimensional transesophageal echocardiogram in the view of the left ventricular outflow tract, showing the tension of both leaflets and the measurement of the posterior leaflet introduced in the clip (red line delimited by plus sings). (**Right**) En-face 3D transesophageal echocardiogram view of the mitral valve with a clip between A2 and P2 with 2 symmetrical pyramids (red dotted lines).

The role of TEE in avoiding this complication is fundamental, it being probably the most important step in performing a meticulous echocardiographic assessment during and after the grasping of the leaflets, ensuring a proper leaflet insertion into the clip arms and valuing the mobility of both leaflets and the amount of their tissue trapped inside the clip [20] (Figure 4). Because the time of this procedure is crucial, it is recommended to acquire a long loop for later visualization and analysis if necessary. 

Recently, the introduction of biplane images has facilitated the assessment of clip attachment due to the acquisition of simultaneous perpendicular views. Different authors have demonstrated that the additional use of 3D-TEE for the assessment of clip attachment may contribute to a reduced rate of subsequent clip complication [3]; therefore, the amount of both leaflets introduced into the clip must be assessed in a 3D enface view observing the amplitude of the inserted tissue and the formation of two pyramids whose vertex must be at least as wide as the clip (Figure 4). Checking a precise rotation of the device by 3D-TEE to avoid asymmetric grasping is another important step to avoid this complication, and, in addition, multiple leaflet grasps may lead to leaflet injury and should be avoided if possible.

**Figure 5:**
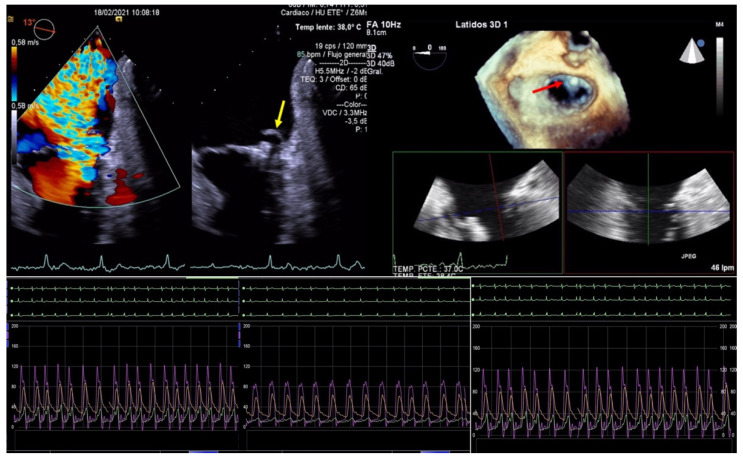
Two-dimensional transesophageal echocardiogram showing a very severe mitral regurgitation after the partial detachment of a clip and a leaflet tear prolapsing in the left atrium (yellow arrow). Three-dimensional transesophageal echocardiogram in the enface mitral view, where a clip can be seen attached to the anterior leaflet (red arrow) and loose from the posterior with a lack of diastolic tissue bridge. In the lower panel, simultaneous recording of left atrium (green), pulmonary artery (yellow), and aortic pressure (purple): baseline, post clip implantation and after detachment.

The echocardiographic criteria to diagnose SLDA are the color Doppler demonstration of significant MR through the leaflet interface, new excessive leaflet mobility following device deployment, and a lack of diastolic tissue bridge by 3DTEE; acute changes in pressure also occur with the new appearance of the v-wave after the initial improvement after clip implantation (Figure 5). Once the complication has occurred, the TEE undoubtedly helps in determining the mechanism of the loosening of the leaflet by measuring the portion of the free leaflet with respect to the measurement prior to implantation [13]. Other mechanisms, such as tear or perforation, can be viewed as a disruption of leaflet integrity reaching the leaflet edge or not, respectively. An excessive clip–leaflet tension can be shown as a shape distortion affecting leaflet coaptation, without disruption of the leaflet integrity. The entrapment of the clip in leaflets or subvalvular structures with chordal rupture appears in TEE as new excessive leaflet mobility [1]. Chordal entanglement should be avoided by minimizing device manipulation below the mitral valve and not advancing the system deep into the left ventricle.

Even though there is scarce information about repeating TEER for recurrent MR, it appears to be a viable approach in inoperable patients, when leaflet insertion into the clip is not compromised. When there is leaflet tear or perforation, repeat percutaneous clip procedures tend to fail [21]. 

#### 4.2.2. Clip Embolization

The complete detachment of a clip from both leaflets, with embolization, is extremely rare (<1%) [1]. This complication is usually observed during the procedure and is recognized immediately. Most of the cases reported required the surgical removal of the clip [6]. 

**Figure 6:**
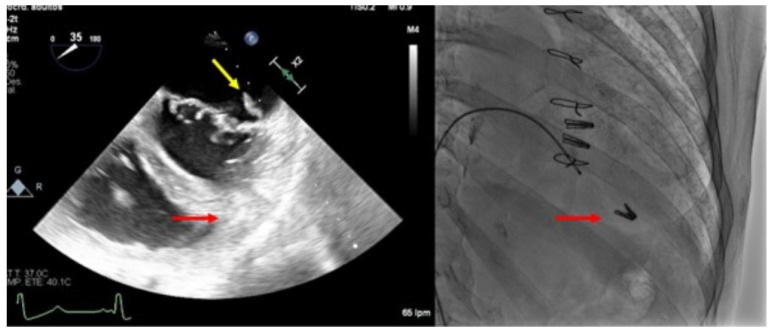
Clip embolization. (**Left**) Two-dimensional transesophageal echocardiogram showing a posterior leaflet prolapse (yellow arrow) after retrograde embolization of a clip to the right ventricle through the procedural atrial septal defect (red arrow). (**Right**) Fluoroscopy image at the end of the procedure showing two clips implanted in the mitral valve and another one embolized in the apex of the right ventricle (red arrow).

Complex mitral anatomy and several-clips implantation with suboptimal echocardiographic window due to the artefacts of the other clips, may be associated with clip embolization (Figure 6). Meticulous intra-procedural imaging with a clear visualization of the device, leaflets, and subvalvular apparatus reduces the risk of detachment.

The treatment of SLDA is generally recommended due to the significant resultant mitral valve regurgitation and the risk of device embolization. The most common way SLDA is managed is by adding additional clips alongside the detached clip [21,22,23]. In addition to treating the mitral regurgitation, this maneuver serves to stabilize the mitral valve leaflets, thereby reducing excessive motion in the region adjacent to the clip and providing direct mechanical contact that stabilizes the SLDA device. In patients with persistent MR and contraindication to surgery, transcatheter electrosurgical laceration of the anterior leaflet and subsequent transcatheter mitral valve implantation has been proposed as an alternative [24].

The treatment of the focal regurgitation has been described using vascular plugs [22], but the risk for further complications and the long-term outcome of this approach is unknown; and, finally, open valve surgery [6,7,21] followed by valve replacement or repair has been successfully reported to treat SLDA.

## 5. Post-TEER Persistent Atrial Septal Defects

Post-TEER persistent atrial septal defects (ASD) have a considerably high incidence, which ranges from 40 to 50% in the available studies [25,26]. Previous studies show inconsistent findings, with some reporting worse clinical outcomes and increased mortality in patients with significant defects [25], while others point to a low clinical impact in the long-term follow-up [26]. Further studies should be conducted to obtain conclusive information on this matter.

Interatrial septal dissection rarely occurs after mitral valve TEER. It consists of a false lumen formation between the mitral valve annular area and the atrial septum, and it might be necessary to treat it with a percutaneous closure device. 

**Figure 7:**
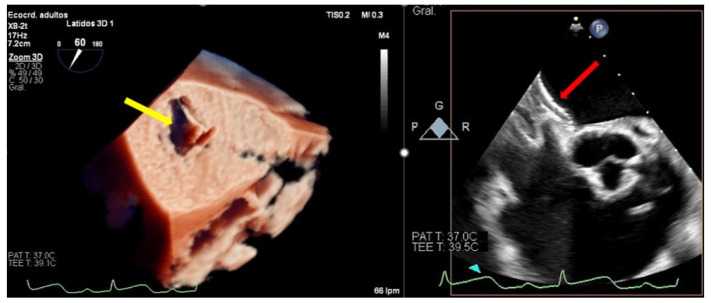
Persistent atrial septal defect. (**Left**) Three-dimensional transesophageal echocardiogram image showing a large atrial septal defect with tearing of the tissue through the transseptal puncture area (yellow arrow) from the left atrium. (**Right**): Two-dimensional transesophageal echocardiogram showing an Amplatzer device closing the iatrogenic atrial septal defect (red arrow).

TEE allows for the assessment of the ASD, morphology, and size, it being superior to visualization through 3D-TEE. If the percutaneous closure of the ASD is necessary, the TEE is essential in guiding the procedure [27] (Figure 7).

## 6. Complications Due to Transesophageal Probe Monitoring

Complications directly owed to TEE monitoring are similar to those described for other procedures, so they are very uncommon, being the most frequent mild oropharyngeal bleeding. However, in a recent study with a large number of patients, the prevalence of TEE-related complications associated with interventional procedures is higher than previously reported. Undergoing a prolonged procedure, particularly in the setting of percutaneous edge-to-edge repair of the mitral valve, was the main factor linked to TEE-related complications, with 7.1% of major complications [28]. 

## 7. Access Site Complications

Venous access for TEER is most commonly performed via the right femoral approach using a 24 F guiding sheath. Vascular access complications may occur due to the immediacy of the femoral vein to the artery and include pseudoaneurysm, arteriovenous (AV) fistula, hematoma requiring transfusion, retroperitoneal hemorrhage, thrombosis, infection, and vessel rupture/perforation [29]. During the post-procedure of TEER, as well as in any interventional procedure using femoral access, unexplained hypotension or falling hemoglobin levels must lead to ruling out vascular access bleeding or retroperitoneal bleeding using imaging testing, mainly CT.

Major vascular access complications often require intervention, such as venous stenting or vascular surgical repair, but their incidence is infrequent. Various studies and registries report rates of 1.4–4% for major and 2.7–3.8% for minor vascular complications [6,7,14,30]. Although the incidence rate is low, preventive measures, such as ultrasound-guided puncture and rapid clinical suspicion, are paramount. The appropriate usage of ultrasound guidance for vascular access improves success rates while reducing iatrogenic injury, the number of needle passes, and infection rates [31]. Regarding venous hemostasis, a recent prospective registry compared the Perclose ProGlide system (Abbott Vascular, Sata Clara, CA, USA) and the figure-of-8 suture following catheter ablation in 434 patients (largest sheath was 15 F). There were no differences in complications between both methods and they improved hemostasis and time to ambulation and permitted more same-day discharge compared to manual compression [32].

## 8. Conclusions

The most frequent major complications of the transcatheter edge-to-edge repair of the mitral valve include tamponade, thromboembolic events, single leaflet device attachment, device embolization, and vascular injury. Since the first use of this procedure in 2003, there have been several advances in the technology and improvements in operator technique, and the incidence of these major complications has decreased over time. Nevertheless, these complications can occur in the best of circumstances, so having a familiarity with the causes and techniques used to manage them will help to ensure optimal outcomes for patients. Meticulous intra-procedural TEE imaging with a clear visualization of the left heart chambers and especially device, leaflets, and subvalvular apparatus reduces the risk of complications during device delivery and deployment in TEER.

## Figures and Tables

**Table 1:** Complications during and after MitraClip implantation.

	EV-I	EV	EV-II	TCVT	GRASP	ACCESS-EU	TRAMI	TVT	COAPT	MITRA FR	Mitra Expand
Type of study	Trial	Trial	Trial	Obs	Obs	Obs	Obs	Obs	Trial	Trial	Obs
Year of publication	2005	2009	2011	2014	2013	2013	2015	2017	2018	2018	2019
Devices generation	1st	1st	1st	1st	1st	1st	1st	1st	1st 2nd	1st 2nd	3rd
Number of patients	27	107	279	628	117	567	828	2952	302	144	107
Complications											
Related to the procedure											
In-hospital death	0%	0.9%	1.0%	2.9%	0.9%	3.4%	2.2%	2.7%	ND	ND	0.9%
Pericardial tamponade	0%	2.8%	1.6%	1.1%	0%	1.1%	1.9%	1%	ND	1.4%	0%
Thromboembolic events ^a^	0%	0.9%	1.0%	0.2%	0.9%	1.1%	0.9%	0.5%	0.7%	1.4%	0%
Acute renal failure	0%	0%	<1.0%	0%	0%	4.8%	0.7%	ND	ND	ND	1%
Major bleeding	3%	3.7%	ND	1.1%	ND	ND	7.4%	3.9%	ND	3.5%	1%
Major vascular complications	0%	ND	1.0%	0.7%	ND	ND	1.4%	1.1%	ND	ND	ND
Related to the clip implantation											
Single-leaflet device attachment	0%	2.8%	5.0%	ND	ND	4.8%	2%	1.5%	ND	ND	4%
Clip embolization	0%	0%	0.0%	0.7%	ND	0%	0%	0.1%	ND	ND	0%
Early partial leaflet detachment ^b^	11%	9%	0.0%	ND	ND	0.2%	2%	ND	ND	ND	0%
Thrombus formation on clip	0%	ND	ND	ND	ND	ND	0.1%	ND	ND	ND	0%
Isolated leaflet damage	0%	ND	ND	ND	ND	ND	ND	ND	ND	ND	2%
Relevant mitral stenosis	0%	ND	0.0%	ND	ND	ND	0.5%	ND	ND	ND	ND
Conversion to open surgery	0%	1.8%	0.0%	0%	0%	0%	0%	0.7%	ND	0%	4%
No procedural success ^c^	3%	26%	23%	4.6%	0%	9%	3.4%	8.2%	2%	4.2%	7%
Cardiac surgery during the first 30 days	3%	0.9%	ND	0%	0%	ND	0.9%	ND	ND	0%	ND

EV: Everest; Obs: observational; ND: no data; ^a^ Thromboembolic events (stroke, myocardial infarction, pulmonary embolism); ^b^ during the procedure or 30 days follow-up; ^c^ operator criteria. Modified from Gheorghe, L. et al (2019) [2].

## Data Availability

Not applicable.
